# Inflammatory fibroid polyp of the renal pelvis: first report at an extra-gastrointestinal site with molecular confirmation

**DOI:** 10.1007/s00428-023-03557-y

**Published:** 2023-05-15

**Authors:** Dora Nagy, Jörg Ellinger, Manuel Ritter, Natalie Pelusi, Glen Kristiansen

**Affiliations:** 1 Institute of Pathology, University Hospital Bonn (UKB), Sigmund-Freud-Str. 25, 53127 Bonn, Germany; 2 Clinic for Urology, University Hospital Bonn (UKB), Bonn, Germany

**Keywords:** Inflammatory fibroid polyp, IFP, Renal pelvis, Spindle cell tumour, *PDFGRA*-mutation

## Abstract

Inflammatory fibroid polyps (IFP) are rare and benign mesenchymal tumours of the gastrointestinal tract. They are submucosal spindle cell lesions with an eosinophilic-rich inflammatory infiltrate and mutations in the platelet-derived growth factor receptor alpha (*PDGFRA*) gene. In this report, we present the case of a 74-year-old female with a solid tumour of the kidney, which presented as a bland proliferation of spindle cells with thin-walled blood vessels and an inflammatory infiltrate with eosinophilic granulocytes. Immunohistochemistry revealed a positivity for vimentin and a weak staining for CD99 and CD34 in the spindle cells. Because of the morphological similarity to IFPs of the gastrointestinal tract, a molecular pathology analysis was performed. This identified an oncogenic mutation in exon 18 of the *PDGFRA* gene, which is characteristic for inflammatory fibroid polyps of the gastrointestinal tract. To the best of our knowledge, this is the first case of an IFP in the urogenital tract.

## Introduction

Inflammatory fibroid polyps (IFP) are known as rare benign mesenchymal tumours of the gastrointestinal tract. This entity was first described in 1949 as a gastric submucosal granuloma with eosinophilic infiltration by Vanek [1]. He reported on various ill-defined submucosal masses located in the stomach, histologically consisting of fibroblasts, collagenous fibres, lymphocytes with occasional rudimentary lymph follicle formation, and eosinophilic granulocytes, with multiple blood and lymph vessels. He already discussed the possibility of a neoplastic process behind these lesions, although he personally considered them rather an inflammatory reaction, and based on Kaijser’s article from 1937, he even suggested a possible allergic cause behind it. In 1953, Helwig et al. [2] suggested the term “inflammatory fibroid polyp” for this entity.

Histologically, inflammatory fibroid polyps are polypoid submucosal lesions which may infiltrate the mucosa. They consist of spindle cells without atypia, and often a so called “onion skin” pattern can be found around blood vessels. The mixed inflammatory infiltrate in the background is commonly rich in eosinophilic granulocytes. On immunohistochemistry, the spindle cells of these tumours typically show a CD34 positivity.

Mainly because of the bland cytology of fibrocytes and fibroblasts and the considerable inflammatory reaction, these lesions were long considered as reactive processes, just as Vanek had originally suggested. In 2008, Huss and Wardelmann et al. described that IFPs harbour mutations in the platelet-derived growth factor receptor alpha (*PDGFRA*) gene [3] and therefore represent true neoplasms of the gastrointestinal tract.

*PDGFRA* is a proto-oncogene and belongs to the receptor tyrosine kinases (RTKs). Besides playing an important role in embryonic development, its role has been described in the pathogenesis of various haematopoietic malignancies [4], glioblastoma [5], and tumours of the gastrointestinal tract, including gastrointestinal stromal tumours [6] and aforementioned inflammatory fibroid polyps [3]. In 2012, Huss et al. [7] reported two cases with *PDGFRA*-exon 14 mutations besides the previously described exon 12 and exon 18 mutations. They suggested two different phenotypes, the “gastric” one which is associated with exon 18 mutations and the “small bowel” type which harbours exon 12 alterations.

Although inflammatory fibroid polyps have been described on various sites of the gastrointestinal tract, even in recent literature reviews [8, 9], the possibility of this entity occurring outside of the alimentary canal has not been considered or examined. To the best of our knowledge, we report here the first case of an IFP in the urogenital tract.

## Case report

**Fig. 1:**
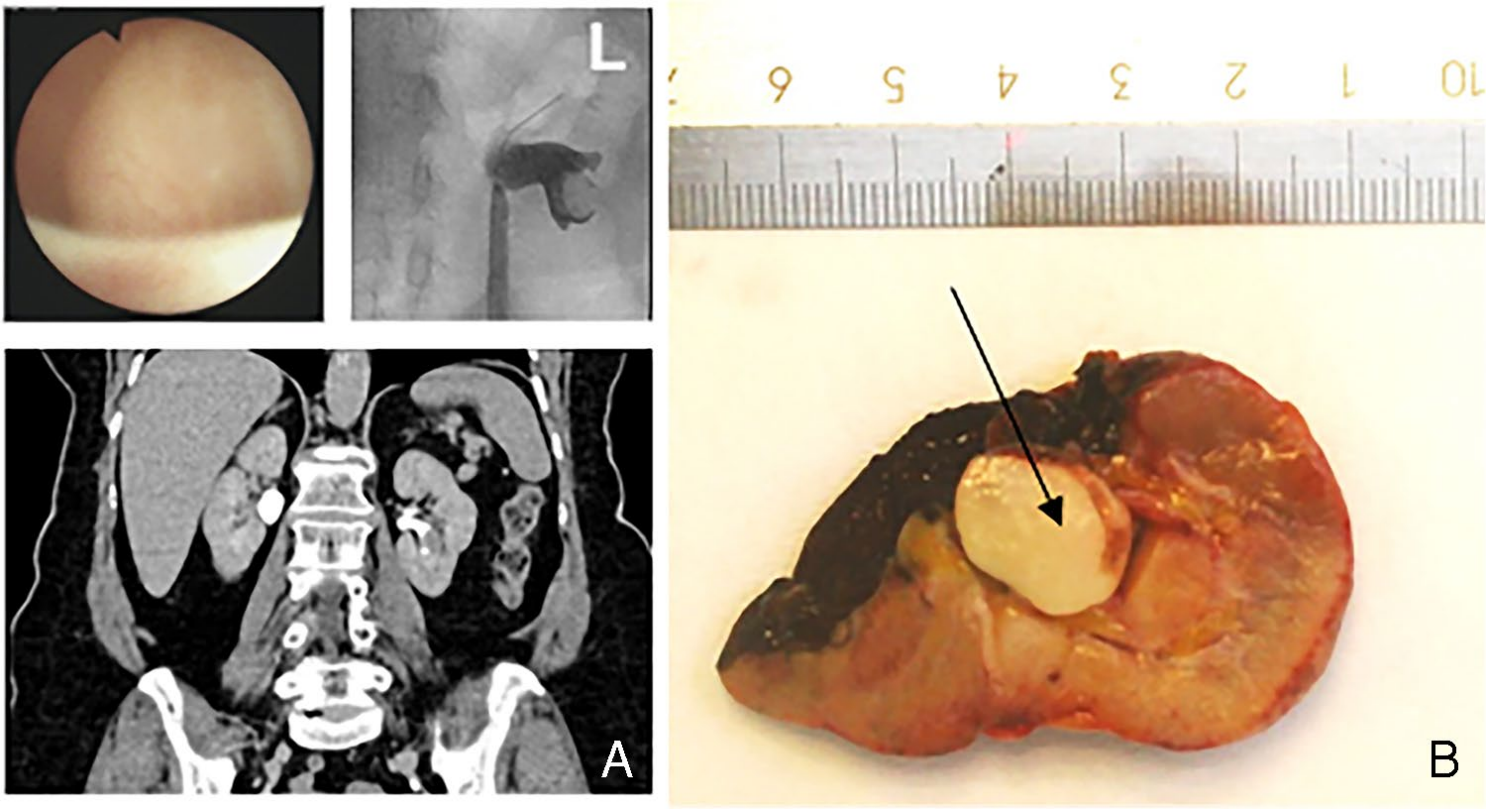
**A** Computed tomography indicated the presence of a tumour in the lower calyx of the left kidney; meanwhile, during diagnostic ureterorenoscopy (upper left corner), a solid tumour was observed in the lower calyx. **B** The tumour (black arrow) macroscopically presented as a white and well-circumscribed mass protruding from the renal pelvis

A 74-year-old female presented with recurrent haematuria; additional micturition disorders were negated. The patient had a history of apoplexy, gastric ulcer, recurrent urolithiasis, hypothyroidism, and arterial hypertension. Family history was without cancer. Urethrocystoscopy excluded the presence of bladder cancer; however, computed tomography indicated the presence of a tumour in the lower calyx of the left kidney. The patient underwent a diagnostic ureterorenoscopy. Endoscopically, a solid tumour was observed in the lower calyx; biopsies were taken; however, both samples did not allow to make a diagnosis due to lack of sufficient tissue. The patient underwent another diagnostic ureterorenoscopy; the tumour was contact vulnerable and bled easily upon touching; thus, it was not possible to obtain enough tissue for pathological examination. As imaging suggested the presence of a renal tumour (Fig. 1a), it was decided to perform an open exploration: a lower pole resection of the left kidney. The frozen section histology suggested a spindle cell tumour, resected with negative surgical margins. The patient was discharged after an uneventful postoperative course.

**Fig. 2:**
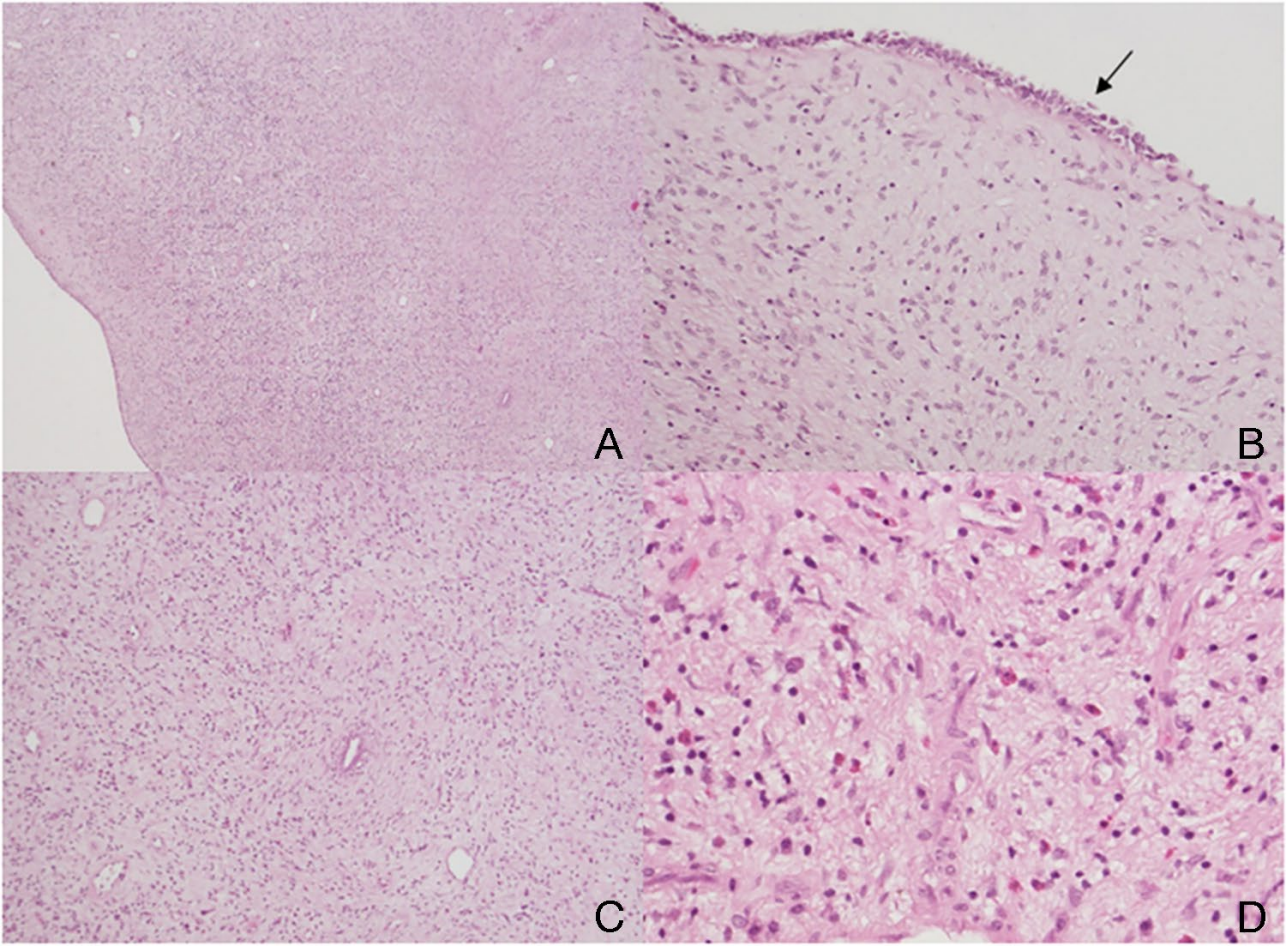
The conventional histomorphology of the tumour shows a well-circumscribed proliferation of bland spindle cells (**A**) beneath a focally denuded urothelial mucosa (**B** urothelium indicated with black arrow). At higher magnification, the cytologically bland tumour cells of the spindle cell neoplasia and dilated blood vessels are discernible (**C**); furthermore, an inflammatory infiltrate with lymphocytes, histiocytes, and eosinophilic granulocytes can also be observed in the background (**D**)

On gross examination, the tumour was a white and well circumscribed, 9 × 13 × 16 mm mass protruding from the renal pelvis (Fig. 1b). The frozen section histology excluded the originally suggested urothelial carcinoma and described a spindle cell neoplasm. On paraffin-embedded tissue sections, a well-circumscribed proliferation of bland spindle cells was observed beneath a focally denuded urothelial mucosa. The tumour cells were cytologically bland, no mitotic figures. Besides thin-walled and dilated blood vessels and entrapped tubuli with reactive changes of the epithelium, the background consisted of an inflammatory infiltrate with lymphocytes, histiocytes, and eosinophilic granulocytes (Fig. 2).

**Fig. 3:**
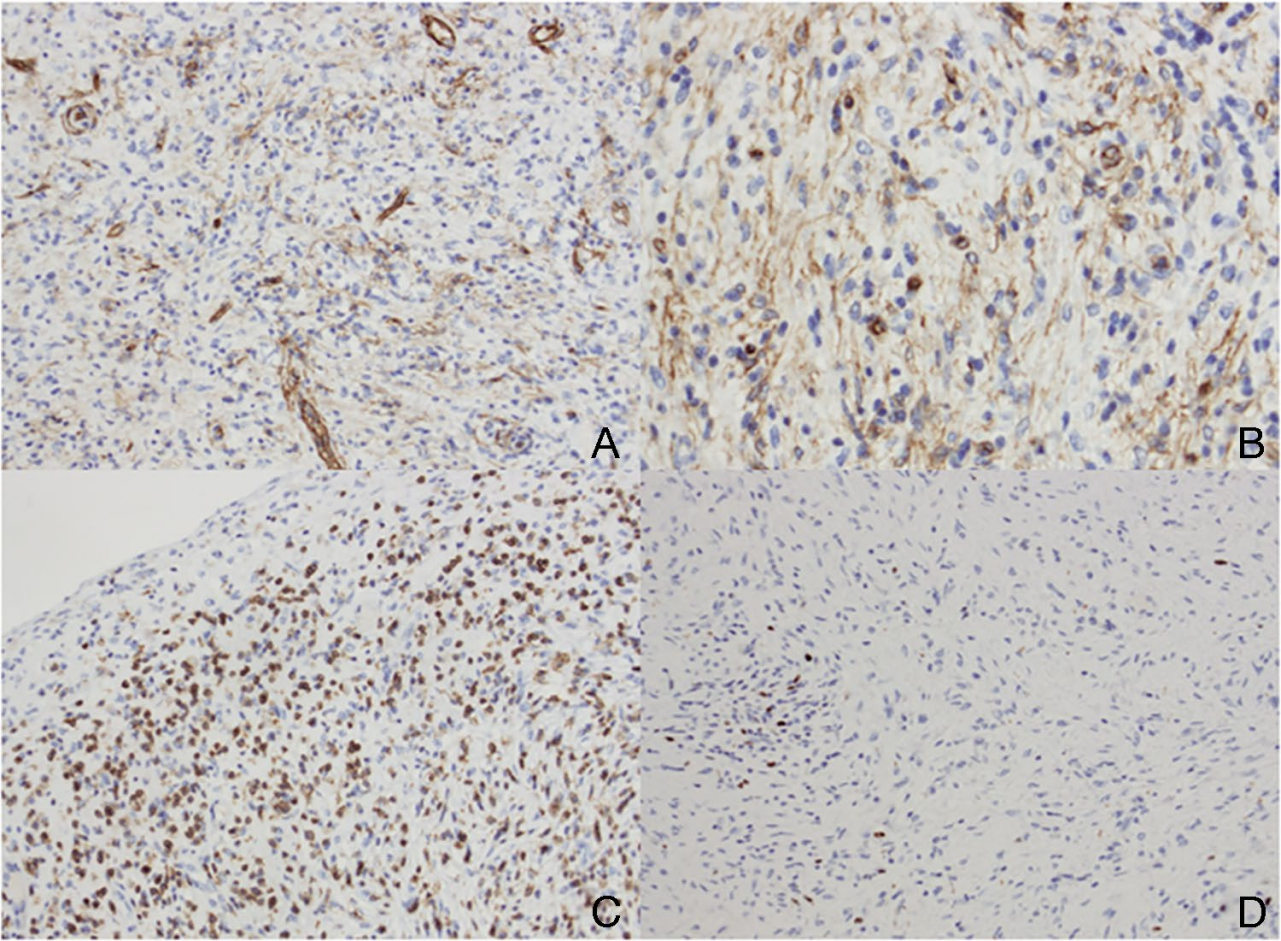
**A, B** The CD34 immunohistochemistry highlights the blood vessels of the tumour and shows a weak to moderate positivity in the spindle cell component of the neoplasia. **C** The LCA antibody accentuates the inflammatory infiltrate in the background of the tumour. **D** The proliferation rate (MIB1-immunohistochemistry) is around 1%, with some hot spots in regions of an enhanced inflammatory component

To determine the entity of the described spindle cell lesion, various immunohistochemical stains have been made. The spindle cells showed a positivity for vimentin and a weak staining for CD99 and CD34. The spindle cells were negative for desmin, S100, ALK1, STAT6, and PDGFRA. SMA and CD34 highlighted the blood vessels and pancytokeratin the entrapped and atrophic tubuli. The proliferation rate (Ki-67) was around 1%, with some hot spots in regions of an enhanced inflammatory component (Fig. 3).

**Fig. 4:**
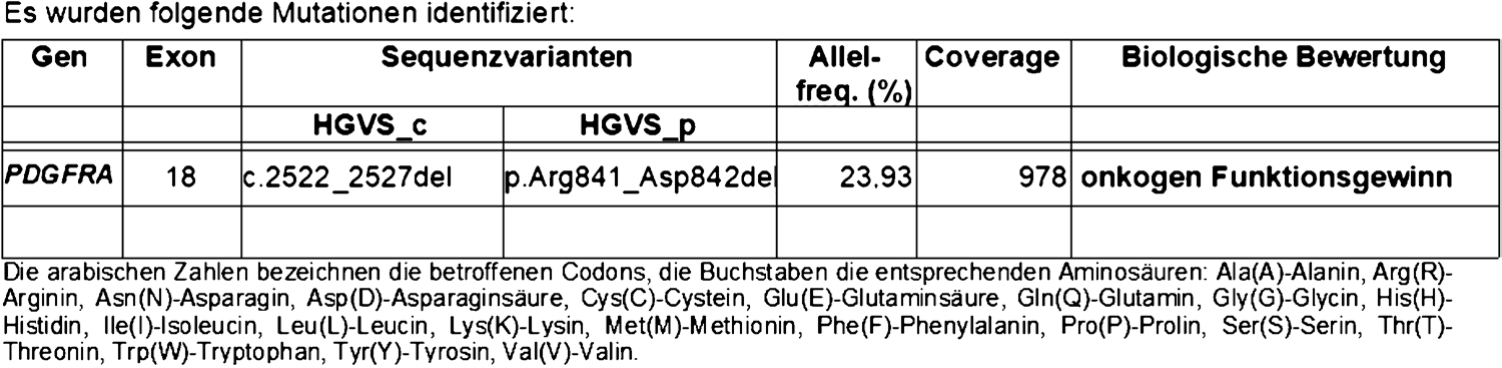
The identified oncogenic mutation in exon 18 of the PDGFRA-gene

The main differential diagnoses were (a) renomedullary interstitial cell tumour (also known as medullary fibroma of the kidney), but this entity is not described as a CD34-positive lesion, or (b) something unusual in this anatomical region. Because of the morphological similarity to IFPs of the gastrointestical tract, a molecular pathology analysis was performed. This identified an oncogenic mutation in exon 18 of the *PDGFRA* gene (Fig. 4), which is characteristic for the inflammatory fibroid polyps of the gastrointestinal tract, stomach type. Accordingly, we signed out this tumour of the renal pelvis as an inflammatory fibroid polyp of the urogenital region.

## Discussion

Inflammatory fibroid polyps have been known as benign mesenchymal tumours for more than 70 years, but their aetiology still remains unclear. To our knowledge, these lesions have so far been described in the gastrointestinal tract, but as we are speaking of a neoplasm originating from stromal cells, there is no clear reason why the causative *PDGFRA* mutation should occur in cells of the gastrointestinal stroma only. As mentioned above, the *PDGFRA* gene plays an important role in the embryonic development as well as the pathogenesis of various haematopoietic malignancies and glioblastomas. Some have reported the presence of IFPs in unusual sites; for example, two cases of inflammatory fibroid polyps were described in the gallbladder. One of these cases is based on conventional-histological and immunohistochemical findings [10], but the other one identified an activating mutation of the *PDGFRA* gene, thus confirming the diagnosis of an inflammatory fibroid polyp [11]. These findings suggest that this entity might occur outside of the luminal gastrointestinal tract; therefore, the question of the possibility of this neoplasm arising on other sites of the body also emerges.

Although the aetiology is still unclear, the identification of a pathogenic mutation in the protooncogen *PDGFRA* supports the neoplastic origin of these mesenchymal lesions. Multiple studies suggested that these polyps are caused by a chemical or physical trigger or trauma [12–14]. The case presented here also supports Virchow’s theory [15] that a chronic inflammation, such as a recurring urolithiasis described here in the renal pelvis, might play a role in the pathogenesis of a neoplasm, specifically an inflammatory fibroid polyp.

## Data Availability

The data that support the findings of this study are openly available upon request.
